# Supplementary material for: The lysosomal transporter MFSD1 is essential for liver homeostasis and critically depends on its accessory subunit GLMP
Source: eLife. 2019 Oct 29;8:e50025. doi: 10.7554/eLife.50025 (PMC6819133; doi:10.7554/eLife.50025)
Supplement: Supplementary file 3. [file elife-50025-supp3.docx]

| **Key Resources Table** | | | | |
| --- | --- | --- | --- | --- |
| **Reagent type (species) or resource** | **Designation** | **Source or reference** | **Identifiers** | **Additional information** |
| Gene (*Mus musculus*) | *Mfsd1* | This paper | GeneID: 66868 | This paper |
| Gene (Mus musculus) | *Glmp* |  | GeneID: 56700 | Mouse line from the lab of Winnie Eskild |
| strain, strain background (*Escherichia coli*) | DH5alpha | Thermo Fisher Scientific |  | Electrocompetent cells |
| Cell Line (*Mus musculus*) | Mouse embryonic fibroblasts  Mfsd1+/+ | This paper |  | Cell line maintained in M. Damme lab; |
| Cell Line (*Mus musculus*) | Mouse embryonic fibroblasts  Mfsd1-/- | This paper |  | Cell line maintained in M. Damme lab; |
| Cell Line (*Mus musculus*) | Mouse embryonic fibroblasts  Glmp+/+ | This paper |  | Cell line maintained in M. Damme lab; |
| Cell Line (*Mus musculus*) | Mouse embryonic fibroblasts  Glmp-/- | This paper |  | Cell line maintained in M. Damme lab; |
| Cell line (*Homo-sapiens*) | Hela | ATCC | RRID:CVCL_0030 |  |
| transfected  construct (*M. musculus*) | MFSD1-HA(pcDNA 3.1) | This paper |  | Transfected construct (mouse) |
| transfected  construct (*M. musculus*) | HA-MFSD1(pcDNA 3.1) | This paper |  | Transfected construct (mouse) |
| transfected  construct (*M. musculus*) | MFSD1 LLAA-HA(pcDNA 3.1) | This paper |  | Transfected construct (mouse) |
| transfected  construct (*M. musculus*) | MFSD1 TD1-HA-TD2(pcDNA 3.1) | This paper |  | Transfected construct (mouse) |
| transfected  construct (*M. musculus*) | MFSD1 LLAA TD1-HA-TD2(pcDNA 3.1) | This paper |  | Transfected construct (mouse) |
| transfected  construct (*M. musculus*) | MFSD1 N76Q-HA(pcDNA 3.1) | This paper |  | Transfected construct (mouse) |
| transfected  construct (*M. musculus*) | MFSD1 N449Q-HA(pcDNA 3.1) | This paper |  | Transfected construct (mouse) |
| transfected  construct (*M. musculus*) | MFSD1 Y195A-HA(pcDNA 3.1) | This paper |  | Transfected construct (mouse) |
| transfected  construct (*M. musculus*) | GLMP-HA(pcDNA 3.1) | This paper |  | Transfected construct (mouse) |
| transfected  construct (*M. musculus*) | GLMP Y400A-HA(pcDNA 3.1) | This paper |  | Transfected construct (mouse) |
| transfected  construct (*M. musculus*) | MFSD1 (pcDNA 3.1) | This paper |  | Transfected construct (mouse) |
| transfected  construct (*M. musculus*) | MFSD1 LLAA(pcDNA 3.1) | This paper |  | Transfected construct (mouse) |
| transfected  construct (*M. musculus*) | GLMP(pcDNA 3.1) | This paper |  | Transfected construct (mouse) |
| transfected  construct (*M. musculus*) | GLMP Y400A(pcDNA 3.1) | This paper |  | Transfected construct (mouse) |
| transfected  construct (*M. musculus*) | LAMP1(pcDNA 3.1) | This paper |  | Transfected construct (mouse) |
| transfected  construct (H*. sapiens*) | LAMP1-HA(pcDNA 3.1) | This paper |  | Transfected construct (mouse) |
| transfected  construct (*M. musculus*) | MFSD1-GFP(pEGFPN1) | This paper |  | Transfected construct (mouse) |
| transfected  construct (*M. musculus*) | MFSD1-GFP(pEGFPN1) | This paper |  | Transfected construct (mouse) |
| transfected  construct (*M. musculus*) | MFSD1 LLAA-GFP(pEGFPN1) | This paper |  | Transfected construct (mouse) |
| transfected  construct (*M. musculus*) | GLMP-GFP(pEGFPN1) | This paper |  | Transfected construct (mouse) |
| transfected  construct (H*. sapiens*) | LAMP1-GFP(pEGFPN1) | This paper |  | Transfected construct (mouse) |
| transfected  construct (*M. musculus*) | MFSD8-GFP(pEGFPN1) | This paper |  | Transfected construct (mouse) |
| transfected  construct (*H. sapiens*) | LAMP1-RFP(pmKATE2) | Fettelschoss et al.,2017 |  | Transfected construct (mouse) |
| transfected  construct (*M. musculus*) | MFSD1-RFP(pmKATE2) | This paper |  | Transfected construct (mouse) |
| transfected  construct (*M. musculus*) | GLMP-RFP(pmKATE2) | This paper |  | Transfected construct (mouse) |
| antibody | anti-MFSD1 (Rabbit polyclonal) | Pineda antibody service |  | IF(1:2500), WB (1:10000) |
| antibody | anti-GLMP (Rabbit Polyclonal) | Pineda antibody service |  | WB(1:500) |
| antibody | anti-CD31  (Rat Monoclonal) | BD Biosciences | Clone MEC 13.3  RRID:AB_2665476 | IF(1:100) |
| antibody | Anti-vWF  (Rabbit polyclonal) | DAKO | A0082 | IF(1:300) |
| antibody | Anti-LAMP2  (Mouse Monoclonal) | Developmental Studies Hybridoma Bank | Clone H4B4, concentrate  RRID:AB_528129 | IF(1:500), WB(1:1000) |
| antibody | Anti-HA-tag(Rat Monoclonal) | Sigma / Roche | Clone 3F10  RRID:AB_2314622 | IF(1:500), WB(1:1000) |
| antibody | POD-coupled anti-HA-tag(Mouse Monoclonal) | Sigma / Roche | Clone 3F10 | WB(1:1000) |
| antibody | PE-coupled anti-HA-tag  (Mouse Monoclonal) | Biolegend | Clone 16B12 | FC(1:400) |
| antibody | Anti-GAPDH  (Rabbit polyclonal) | Santa Gruz | FL-335  RRID:AB_10167668 | WB(1:2000) |
| antibody | Anti-ATPase  (Mouse Monoclonal) | Millipore | Clone 464.6 | WB(1:1000) |
| antibody | Anti-LAMP1 (Rat Monoclonal) | Developmental Studies Hybridoma Bank | Clone 1D4B, concentrate  RRID:AB_2134500 | IF(1:500), WB(1:1000) |
| antibody | Anti-GM130  (Mouse Monoclonal) | BD Biosciences | Clone35/GM130  RRID:AB_398141 | IF(1:500), WB(1:1000) |
| antibody | Anti-KDEL  (Mouse Monoclonal) | Enzo | Clone 10C3  RRID:AB_2039327 | IF(1:500), WB(1:1000) |
| antibody | Anti-Integrin α-IIb  (Rat Monoclonal) | BioLegend | Clone MW Reg30 | IF(1:1000) |
| antibody | Anti-VDAC  (Rabbit polyclonal) | Sigma | **V2139**  RRID:AB_477610 | WB(1:2000) |
| antibody | Anti-tubulin  (Mouse Monoclonal) | Developmental Studies Hybridoma Bank | Clone E7  RRID:AB_528499 | WB(1:1000) |
| antibody | Anti-F4/80  (Rat Monoclonal) | Austyn and Gordon 1981 |  | IF(1:500) |
| antibody | Actin,TR-phalloidin | Life technologies | T7471 | IF(1:300) |
| Peptide, recombinant protein | Endoglycosidase H | Sigma Aldrich | 11088726001 |  |
| Peptide, recombinant protein | PNGaseF | Sigma Aldrich | 11365177001 |  |
| Sequence-based reagent | CD34_F | This Paper | qPCR primers | gggtagctctctgcctgatg |
| Sequence-based reagent | CD34_R | This Paper | qPCR primers | tccgtggtagcagaagtcaa |
| Sequence-based reagent | CXCL1_F | This Paper | qPCR primers | gactccagccacactccaac |
| Sequence-based reagent | CXCL1_R | This Paper | qPCR primers | tgacagcgcagctcattg |
| Sequence-based reagent | MCP1_F | This Paper | qPCR primers | catccacgtgttggctca |
| Sequence-based reagent | MCP1_R | This Paper | qPCR primers | gatcatcttgctggtgaatgagt |
| Sequence-based reagent | MMP2_F | This Paper | qPCR primers | aactttgagaaggatggcaagt |
| Sequence-based reagent | MMP2_R | This Paper | qPCR primers | tgccacccatggtaaacaa |
| Sequence-based reagent | MMP9_F | This Paper | qPCR primers | acgacatagacggcatcca |
| Sequence-based reagent | MMP9_R | This Paper | qPCR primers | gctgtggttcagttgtggtg |
| Sequence-based reagent | F8_F | This Paper | qPCR primers | agatacacttaccctgttcccatt |
| Sequence-based reagent | F_R | This Paper | qPCR primers | accccaagacccatagacct |
| Sequence-based reagent | GLMP_F | This Paper | qPCR primers | agtgaacgaacggaactcca |
| Sequence-based reagent | GLMP_R | This Paper | qPCR primers | tggagaagagccccataaca |
| Sequence-based reagent | MFSD1_F | This Paper | qPCR primers | tcgaagggatacttgctgct |
| Sequence-based reagent | MFSD1_R | This Paper | qPCR primers | gcacggttcaccaagtacag |
| Sequence-based reagent | Mouse GAPDH endogenous control | Thermofisher | 4352932E |  |
| Sequence-based reagent | Cre Fw | This Paper | Genotyping primers | atgcgctgggctctatggcttctg |
| Sequence-based reagent | Cre Rv | This Paper | Genotyping primers | tgcacacctccctctgcatgcacg |
| Sequence-based reagent | Flp Fw | This Paper | Genotyping primers | gtcactgcagtttaaatacaagacg |
| Sequence-based reagent | Flp Rv | This Paper | Genotyping primers | gttgcgctaaagaagtatatgtgcc |
| Sequence-based reagent | MFSD1-F | This Paper | Genotyping primers | tatggactctgcccacagtgttacg |
| Sequence-based reagent | MFSD1-R | This Paper | Genotyping primers | aatggccaaagacaggcagaaatgg |
| Sequence-based reagent | MFSD1-ttR | This Paper | Genotyping primers | actcagcccttttctgtctcctacg |
| Sequence-based reagent | neoF | This Paper | Genotyping primers | gggatctcatgctggagttcttcg |
| Sequence-based reagent | mMfsd1_HindIII_N-HA | This Paper | Cloning primers | gtataagcttatggcttacccatacgacgtcccagactacgctgaggacgaggatggggaagat |
| Sequence-based reagent | mMfsd1_XbaI_N-HA | This Paper | Cloning primers | gatctctagattactctggatgagagagctt |
| Sequence-based reagent | mMfsd1_HindIII_C-HA | This Paper | Cloning primers | gtataagcttatggaggacgaggatggggaa |
| Sequence-based reagent | mMfsd1_XbaI_C-HA | This Paper | Cloning primers | gatctctagattaagcgtagtctgggacgtcgtatgggtactctggatgagagagcttcat |
| Sequence-based reagent | mMfsd1_BamHI_C-GFP | This Paper | Cloning primers | gatcggatccccctctggatgagagagcttcat |
| Sequence-based reagent | MFSD1 (TD1-HA-TD2) Rv | This Paper | Cloning primers | agcgtagtctgggacgtcgtatgggtagtcccgtttcacctgagtct |
| Sequence-based reagent | MFSD1 (TD1-HA-TD2) Fw | This Paper | Cloning primers | tacccatacgacgtcccagactacgctatgcaagtgaacaccacgaa |
| Sequence-based reagent | MFSD1 HindIII Fw | This Paper | Cloning primers | gtataagcttatggaggacgaggatggggaa |
| Sequence-based reagent | MFSD1 XhoI Rv | This Paper | Cloning primers | aagactcgagttactctggatgagagagct |
| Sequence-based reagent | GLMP HindIII Fw | This Paper | Cloning primers | gtataagcttatgtttcgctgttggggacct |
| Sequence-based reagent | GLMP XbaI C-HA Rv | This Paper | Cloning primers | gatctctagattaagcgtagtctgggacgtcgtatgggtagtttatggactggtactcaga |
| Sequence-based reagent | GLMP XhoI Rv | This Paper | Cloning primers | gatcctcgagtcagtttatggactggtact |
| Sequence-based reagent | GLMP Y400A XhoI Rv | This Paper | Cloning primers | gatcctcgagtcagtttatggactgggcct |
| Sequence-based reagent | GLMP_EcoRI Fw | This Paper | Cloning primers | atcggaattcatgtttcgctgttggggacct |
| Sequence-based reagent | mGLMP BamHI Rv | This Paper | Cloning primers | gatcggatccccgtttatggactggtactcaga |
| Sequence-based reagent | LAMP1 HindII Fw | This Paper | Cloning primers | gatcaagcttatggcggcccccggcgcccgg |
| Sequence-based reagent | LAMP1 XbaI Rv | This Paper | Cloning primers | gatctctagactagatggtctgatagccggc |
| Sequence-based reagent | hLAMP1 HindIII Fw | This Paper | Cloning primers | gcgcccaagcttatggcggcccccggcagcgcc |
| Sequence-based reagent | hLAMP1 C-HA XhoI Rv | This Paper | Cloning primers | gcgcccctcgagttaagcgtagtctgggacgtcgtatgggtagatagtctggtagcctgcgtg |
| Sequence-based reagent | MFSD8 BglII Fw | This Paper | Cloning primers | gatcgagatctatggcgaacctgggaagt |
| Sequence-based reagent | MFSD8 HindIII Rv | This Paper | Cloning primers | gatcgaagcttctcctggatcctcatata |
| Sequence-based reagent | Mfsd1_Y195A_for | This Paper | Mutagenesis primers | taatgcttcaatcttcccagccagccatcccatgaggttc |
| Sequence-based reagent | Mfsd1_Y195A_rev | This Paper | Mutagenesis primers | gaacctcatgggatggctggctgggaagattgaagcatta |
| Sequence-based reagent | Mfsd1_L11A/L12A_for | This Paper | Mutagenesis primers | ggaagatcgggcggcggctgggggccgccgcg |
| Sequence-based reagent | Mfsd1_L11A/L12A_rev | This Paper | Mutagenesis primers | cgcggcggcccccagccgccgcccgatcttcc |
| Sequence-based reagent | Mfsd1_N76Q_for | This Paper | Mutagenesis primers | gaaacgggacatgcaagtgcagaccacgaaattcatgctgc |
| Sequence-based reagent | Mfsd1_N76Q_rev | This Paper | Mutagenesis primers | gcagcatgaatttcgtggtctgcacttgcatgtcccgtttc |
| Sequence-based reagent | Mfsd1_N449Q_for | This Paper | Mutagenesis primers | gtgcccaaggagggaatctacagtattctgcaaaacaaaggga |
| Sequence-based reagent | Mfsd1_N449Q_rev | This Paper | Mutagenesis primers | tccctttgttttgcagaatactgtagattccctccttgggcac |
